# Laser-driven shock compression of “synthetic planetary mixtures” of water, ethanol, and ammonia

**DOI:** 10.1038/s41598-019-46561-6

**Published:** 2019-07-12

**Authors:** M. Guarguaglini, J.-A. Hernandez, T. Okuchi, P. Barroso, A. Benuzzi-Mounaix, M. Bethkenhagen, R. Bolis, E. Brambrink, M. French, Y. Fujimoto, R. Kodama, M. Koenig, F. Lefevre, K. Miyanishi, N. Ozaki, R. Redmer, T. Sano, Y. Umeda, T. Vinci, A. Ravasio

**Affiliations:** 1 LULI, CNRS, CEA, École Polytechnique, Institut Polytechnique de Paris, route de Saclay, 91128 Palaiseau cedex, France; 2 Sorbonne Université, Faculté des Sciences et Ingénierie, Laboratoire d’utilisation des lasers intenses (LULI), Campus Pierre et Marie Curie, place Jussieu, 75252 Paris cedex 05, France; 3 Institute for Planetary Materials, Okayama University, Misasa, Tottori, 682-0193 Japan; 4 GEPI, Observatoire de Paris, PSL Université, CNRS, 77 avenue Denfert Rochereau, 75014 Paris, France; 5 Universität Rostock, Institut für Physik, 18051 Rostock, Germany; 6 Graduate School of Engineering, Osaka University, Suita, Osaka, 565-0871 Japan; 7 Open and Transdisciplinary Research Initiatives, Osaka University, Suita, Osaka, 565-0871 Japan; 8 Institute of Laser Engineering, Osaka University, Suita, Osaka, 565-0871 Japan

**Keywords:** Exoplanets, Giant planets, Chemical physics, Laser-produced plasmas

## Abstract

Water, methane, and ammonia are commonly considered to be the key components of the interiors of Uranus and Neptune. Modelling the planets’ internal structure, evolution, and dynamo heavily relies on the properties of the complex mixtures with uncertain exact composition in their deep interiors. Therefore, characterising icy mixtures with varying composition at planetary conditions of several hundred gigapascal and a few thousand Kelvin is crucial to improve our understanding of the ice giants. In this work, pure water, a water-ethanol mixture, and a water-ethanol-ammonia “synthetic planetary mixture” (SPM) have been compressed through laser-driven decaying shocks along their principal Hugoniot curves up to 270, 280, and 260 GPa, respectively. Measured temperatures spanned from 4000 to 25000 K, just above the coldest predicted adiabatic Uranus and Neptune profiles (3000–4000 K) but more similar to those predicted by more recent models including a thermal boundary layer (7000–14000 K). The experiments were performed at the GEKKO XII and LULI2000 laser facilities using standard optical diagnostics (Doppler velocimetry and optical pyrometry) to measure the thermodynamic state and the shock-front reflectivity at two different wavelengths. The results show that water and the mixtures undergo a similar compression path under single shock loading in agreement with Density Functional Theory Molecular Dynamics (DFT-MD) calculations using the Linear Mixing Approximation (LMA). On the contrary, their shock-front reflectivities behave differently by what concerns both the onset pressures and the saturation values, with possible impact on planetary dynamos.

## Introduction

Mixtures of light elements at extreme pressure and temperature conditions exhibit intriguing chemical and physical processes, involving complex bonding configurations. Of particular interest are mixtures of water (H_2_O), methane (CH_4_), and ammonia (NH_3_), also called planetary “ices”, as they comprise the major components of the interiors of the ice giant planets Uranus and Neptune. It is commonly assumed that they consist of an outer hydrogen-helium layer, an “icy” mantle, and possibly a rocky core^1–3^. As pressure and temperature increase from the outer layers towards the core, icy mixtures are expected to lose their original molecular nature and to exhibit a wide range of different states embracing atomic, molecular, and dissociated fluids, plasmas, and superionic lattices^4,5^. In more detail, the term “ice” refers to unspecified high-pressure phases of mixtures whose exact composition is unclear as discussed in previous work^2,3^.

The complex behaviour of these mixtures at planetary interior conditions – pressures of several megabar (1 Mbar = 100 GPa) and temperatures of a few thousand Kelvin – is at the basis of several gaps in our understanding of Uranus and Neptune. Their internal structures are inferred from the observed gravitational fields, masses, rotational velocities, and radii. However, their mass distributions remain ambiguous, especially in their deep interiors^3,6^. An accurate analysis of the Voyager 2 data^7–10^ even opens the possibility for a dichotomy in their structures, indicating that the two planets could have different internal structures and thermal histories despite being similar in mass and radius. Indeed, the data suggest an internal heat source for Neptune, while Uranus’ low luminosity indicates that it is presently in thermal equilibrium with the radiation received from the Sun^11,12^. Moreover, our lack of precise information on transport properties of the planetary ices such as conductivities is casting serious issues in explaining Uranus and Neptune’s non-dipolar, non-axisymmetric magnetic fields^13^. While it is customarily assumed that their peculiar structure is due to the fact that they originate in a thin outer shell, it is not clear if the main contribution to the planetary dynamo is given by hydrogen, which is present in the atmosphere and becomes metallic at around 1 Mbar^3,14^, or by the icy mixture in the deep interiors^15,16^. Resolving this situation is even more urgent today, given that the number of known exoplanets keeps growing steadily with lots of them similar in size and radius to Uranus and Neptune. Since solar planets are used as prototypes for extrasolar planets^17^, the loose description of planetary ices and the resulting approximate portrait of Uranus and Neptune not only prevent the understanding of extrasolar giant planets such as GJ 436 b or HAT-P-11b but also affects our capability to distinguish Earth-like candidates. As a result, there is a great need to establish benchmarking values for the equations of states, phase diagrams, and transport properties of H_2_O-CH_4_-NH_3_ mixtures at megabar pressures and temperatures of a few thousand Kelvin.

So far, our knowledge of icy mixtures relies on Density Functional Theory Molecular Dynamics (DFT-MD) calculations and on few experimental data. DFT-MD calculations have provided equations of state, pair distribution functions, and bond autocorrelation functions^5,18–20^, allowing to deduce the structural (such as compressibility) and transport (such as viscosity) properties of the icy mixtures. The available experimental works have primarily characterised the equation of state and electrical conductivity, using single-shock compression – thus exploring thermodynamic conditions along the principal Hugoniot curve^5,21,22^, or double-shock compression, which allowed to probe lower temperatures^5,22^. However, existing experimental data are limited to 0.8 Mbar along the principal Hugoniot curve and 2.2 Mbar off the Hugoniot, missing temperature and reflectivity measurements. Pure water, on the contrary, has been compressed up to higher pressures^23–29^, along the principal, statically pre-compressed, and the ice VII Hugoniot curves. Moreover, the water shock-front reflectivity has been measured in the metallic regime^25–28^, whereas similar data are not available for icy mixtures.

In the present work, using laser-driven shocks we have compressed pure water, a water-ethanol mixture (WEM), and a water-ethanol-ammonia “synthetic planetary mixture” (SPM) representative for the interiors of Uranus and Neptune and similar to the previously studied “synthetic Uranus”^22^. We have measured the thermodynamic state of the shocked sample and the optical reflectivity of the shock front up to a pressure of 2.6 Mbar using standard rear-side optical diagnostics: two Doppler velocity interferometers (VISAR), a VISAR-independent reflectivity measure, and a streak optical pyrometer (SOP).

## Methods and Experimental Setup

Pure water, a water-ethanol mixture, and a water-ethanol-ammonia mixture have been studied in order to characterise the behaviour of mixtures of various compositions and to isolate possible effects due to different chemical environments linked to the presence of carbon and nitrogen atoms. The water-ethanol mixture (WEM) is composed of 49.45% pure water and 50.55% pure ethanol in mass ratios, which correspond to the atomic abundance ratios of H:C:N:O = 22:4:0:7. The water-ethanol-ammonia synthetic planetary mixture (SPM) has been prepared by adding up pure water, pure ethanol, and a liquid water-ammonia (28% wt.) mixture with the following mass ratios: 23.26% of water, 46.23% of ethanol, 30.51% of water-ammonia solution. The following atomic ratio has been obtained: H:C:N:O = 25:4:1:7, with the aim of reproducing the chemical composition of Uranus’ and Neptune’s mantles. The C:N:O abundance ratios are comparable to those of the Solar System^30^. Ethanol has been preferred over methane as component of the mixture to avoid demixing issues. The refractive index at 532 nm has been measured for both mixtures as ${n}_{0}^{{\rm{S}}{\rm{P}}{\rm{M}},{\rm{W}}{\rm{E}}{\rm{M}}}\,=1.36$. The values at 1064 nm have been extrapolated using the same wavelength dependence of the refractive index as that of water. The densities of the mixtures at ambient conditions are ${\rho }_{0}^{{\rm{SPM}}}=0.889$ g/cm^3^ and ${\rho }_{0}^{{\rm{WEM}}}=0.881$ g/cm^3^.

Experiments were performed at the GEKKO XII laser facility (Institute of Laser Engineering, Osaka University, Japan)^31^ and at the LULI2000 laser facility (École Polytechnique, France). At GEKKO XII, 3 up to 9 beams (corresponding to energies on target from 120–440 J) at 351 nm have been used, with a 600 *μ*m focal spot diameter. At LULI2000, 1 or 2 beams (energies on target from 200–500 J) at 527 nm have been used, with a 500 *μ*m focal spot diameter. In both cases, the laser pulse duration was 2.5 ns and phase plates were used to obtain a uniform irradiation spot. As rear-side probe lasers, a YAG at 532 nm was used at GEKKO XII with a full-width half-maximum pulse duration of ~10 ns and a YAG at 532 and 1064 nm was used at LULI2000, with a full-width half-maximum pulse duration of ~20 ns.

To optimise the target design and ensure there were no shock reverberations in the sample, the laser-target interaction and the shock loading into the cell have been simulated with the Lagrangian 1-D hydrodynamic code MULTI^32^. The equation of state of the target components have been extracted from the SESAME tables^33,34^. The table 7154 for water has been used for the mixtures. The multi-layered target cells were composed of a 10–15 *μ*m thick polystyrene ablator, a 40 *μ*m thick aluminium shield, a 50 *μ*m thick *α*-quartz standard, the sample (4 mm thick), and a rear *α*-quartz window (200 *μ*m thick). We also performed some high-intensity shots at GEKKO XII with 50 *μ*m of polystyrene/3 *μ*m of gold/5 *μ*m of aluminium/20 *μ*m of *α*-quartz/4 mm of sample/200 *μ*m of *α*-quartz targets. The gold layer served as X-ray shield to prevent any pre-heating of the sample. Detailed information about the target cells can be found in the Supplementary information.

A decaying shock technique has been employed. In this approach, the laser pulse is designed to produce a strong shock that decays in time. As the shock propagates in the sample, each successive layer is compressed at a different pressure-temperature condition on the principal Hugoniot curve. As long as the optical depth of the loaded state is lower than the longitudinal temperature gradient length scale, the collected self-emission can be considered as originating from a thin layer behind the shock front, thus allowing a temperature measurement of the Hugoniot states loaded by the shock^35,36^.

The time-resolved shock velocity *U*_*s*_(*t*) starting from the shock arrival time in the quartz sample $t={t}_{1}$ has been extracted using the Neutrino software^37^ from the output of the two VISARs^38^ (see the Supplementary information), both working at 532 nm (GEKKO XII) or working one at 532 and one at 1064 nm (LULI2000). The VISAR velocity-per-fringe parameters were 4.476 and 7.432 km/s at GEKKO XII, 15.94 and 6.08 km/s at LULI2000. The thermodynamic conditions (the mass density *ρ*, the pressure *p*, and the internal energy density *E*) reached in the mixture at the moment of the shock arrival have been obtained from the Rankine-Hugoniot relations^39,40^ through impedance mismatch^41^, using quartz as in-situ standard. Impedance mismatch requires the measure of the shock velocities (${U}_{s}^{{\rm{Qz}}}$ and ${U}_{s}^{{\rm{sample}}}$ right before and after the time $t={t}_{2}$ when the shock crosses the quartz/mixture interface. We were able to extract ${U}_{s}^{{\rm{Qz}}}$ and ${U}_{s}^{{\rm{sample}}}$ from the time-dependent shock velocity *U*_*s*_(*t*) via an extrapolation to *t*_2_ of a linear fit on a 1–2 ns time window ending 100–200 ps before (for ${U}_{s}^{{\rm{Qz}}}$) or beginning 100–200 ps after (for ${U}_{s}^{{\rm{sample}}}$) *t*_2_. Shock versus fluid (or particle) velocity (*U*_*s*_-*U*_*p*_) data from the Sandia Z-pinch facility^42^ have been used as reference for quartz. The adiabatic release of quartz on the lower-impedance mixture has been approximated using the mirror reflection of the quartz Hugoniot with respect to the line ${U}_{p}={U}_{p}^{Qz}$ in the *p*-*U*_*p*_ plane. This approximation is validated since in this regime the entropy increase along a Hugoniot curve differs from an adiabatic path only at the third order in relative compression. We verified that this approximation agrees within the error bars with a Mie-Grüneisen release model^43^ in the region where the latter can be applied.

**Figure 1:**
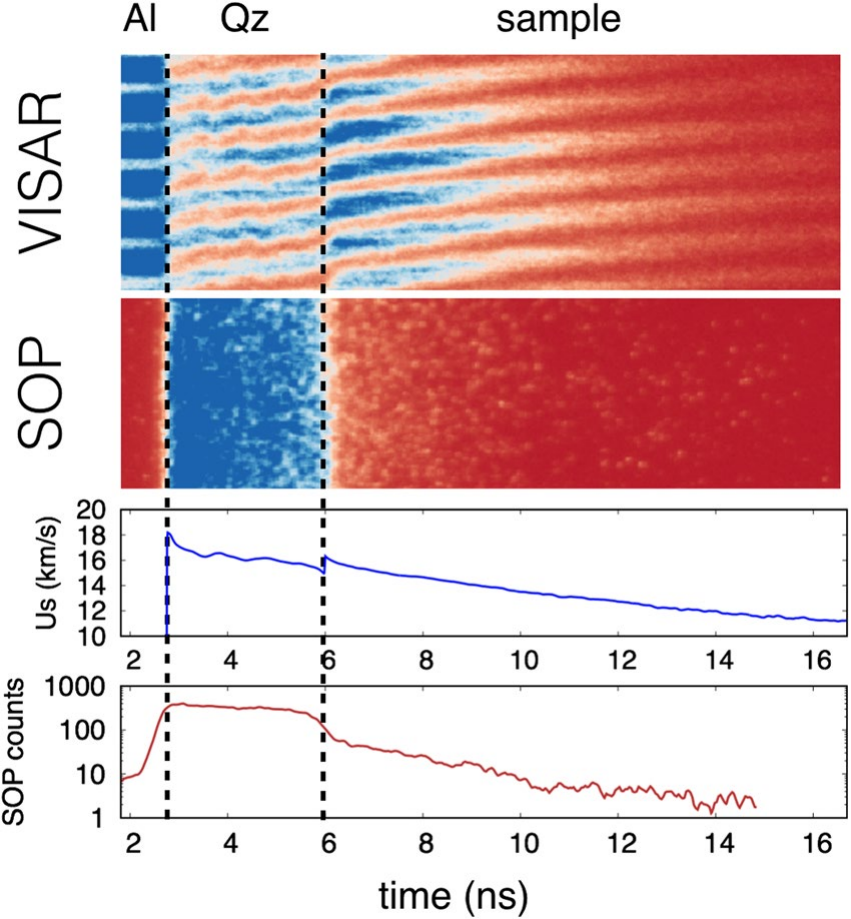
Diagnostics output of a typical shot on the SPM at the GEKKO facility. From top to bottom: VISAR image; SOP image; time-resolved shock velocity; time-resolved SOP counts. The three time intervals indicate when the probe laser is reflected by aluminium (Al), when a reflecting shock front is propagating through the quartz layer (Qz), and when the shock front is propagating through the mixture sample. The transverse target dimension is ~180 *μ*m.

The time-resolved self-emission has been measured through a streaked optical pyrometer (SOP) working in a spectral range centred around ${\lambda }_{{\rm{S}}{\rm{O}}{\rm{P}}}=455\,{\rm{n}}{\rm{m}}$ with a FWHM of 20 nm. The time-resolved temperature of the states reached downstream the decaying shock has been obtained from Planck’s law $T(t)={T}_{0}/\,{\rm{l}}{\rm{n}}[1+A{\varepsilon }_{532{\rm{n}}{\rm{m}}}\,(t)/{N}_{c}(t)]$, where ${T}_{0}=hc/{k}_{B}{\lambda }_{{\rm{S}}{\rm{O}}{\rm{P}}}$, *A* is a calibration factor, ${\varepsilon }_{455{\rm{n}}{\rm{m}}}\,(t)$ is the time-resolved emissivity of the shock front at the working wavelength and *N*_*c*_(*t*) is the time-resolved number of counts on the SOP. To get the emissivity at 455 nm we used the reflectivity measured at 532 nm under a grey-body hypothesis: ${\varepsilon }_{455{\rm{n}}{\rm{m}}}\,(t)=1-{R}_{532{\rm{n}}{\rm{m}}}\,(t)$. This hypothesis introduces a negligible error source, since the temperature measures depend logarithmically on the emissivity and, as the measured reflectivity was often below 30%, a relative change in reflectivity is attenuated when $\varepsilon =1-R$ is calculated. The SOP calibration has been made either in situ, by determining the *A* factor using quartz as standard (GEKKO XII), or using a calibration lamp with known emission temperature (LULI2000, see the Supplementary information). A typical VISAR and SOP output is shown in Fig. 1, together with the extracted shock velocity and self-emission temporal profiles.

The shock compression of the samples induces a refractive index change. According to the Fresnel equations, this implies that the shock front will reflect a fraction of the incident light from the probe laser. The time-resolved reflectivity of the shock front has been measured with the VISARs as the ratio between the shot signal and a reference signal reflected on the aluminium/quartz interface and normalised using the known relation between shock velocity and reflectivity in quartz at 532 nm^44^ and 1064 nm^45^. A time-resolved VISAR-independent reflectivity measurement at 532 nm (“reflectometer”) was included in the setup to corroborate the reflectivity measurements obtained from the VISAR fringe system.

The Hugoniot curves of the WEM and the SPM are reproduced by DFT-MD calculations using a Linear Mixing Approximation (LMA). The LMA is performed using the pure water, methane, and ammonia equations of state from DFT-MD simulations^20^. In particular, the WEM is modelled by a 7:4 water-methane mixture, while the SPM is represented by a 7:4:1 water-methane-ammonia mixture. A quantum correction to the internal energies has been added as motivated in previous work^46^. This correction typically yields much better agreement between simulation and experiment along the Hugoniot as has been shown for pure ammonia^47^. Since the stoichiometry of the mixtures in the simulations differs slightly from the experiment, the initial Hugoniot densities have been determined by performing additional DFT-MD calculations employing VASP^48–50^. Four to six density points have been computed along the 298 K isotherms for water, methane, ammonia, the 7:4 water-methane mixture, and the 7:4:1 water-methane-ammonia mixture. For the pure compounds, the same parameters as in a previous work^20^ have been used, while 88 and 96 molecules have been used for the binary and ternary mixture, respectively. The linear mixing approximation is found to agree within ±2 kJ/g in energy for both mixtures along the 298 K isotherm. In linear mixing densities differ from the real mixtures up to 6% and 3% for the binary and ternary mixture, respectively. Overall, these findings are within the range discussed in a previous work^20^, except the 6% discrepancy in density for the water-methane mixture, which is most likely due to well-known pressure-dependent solubility for this mixture^51^. Finally, initial densities of 0.7019 g/cm^3^ for the water-methane mixture and 0.7301 g/cm^3^ for the water-methane-ammonia mixture have been found at the pressures given by the experimentally used real mixtures containing ethanol.

## Results and Discussion

### Equation of state

**Figure 2:**
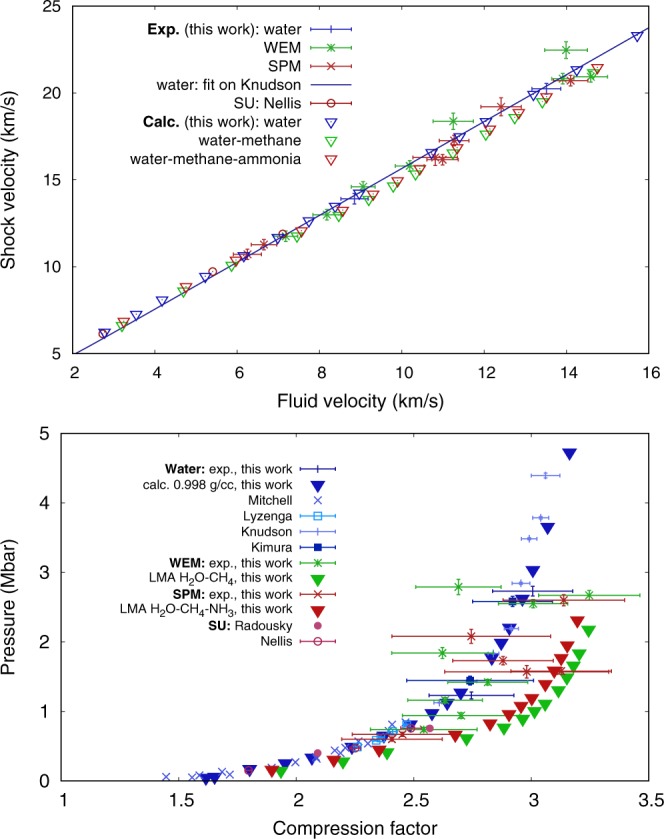
Top. Experimental shock - fluid velocity data on water, WEM, and SPM (blue, green, and red points with error bars, respectively) compared to DFT-MD calculations (triangles). A linear fit on previous water data^26^ and previous experimental data on synthetic Uranus^22^ (similar to the SPM) are shown for comparison. Bottom. Transposition in the pressure - compression factor (*ρ*/*ρ*_0_) plane of the data displayed on the top image. Other previous data on water^23,24,27^ and on synthetic Uranus^21^ are shown.

Figure 2 (top) shows the *U*_*s*_-*U*_*p*_ data for pure water, the WEM, and the SPM, together with previous results on the similar “synthetic Uranus”^22^. A linear fit on previous water data^26^, *U*_*s*_ = (1.350 ± 0.014) ${U}_{p}+(2.16\pm 0.15)\,{\rm{km}}/{\rm{s}}$ is also shown. The corresponding figure for all available data on pure water is provided in the Supplementary information. The two water data points of this study are in perfect agreement, thus validating the experimental procedure. The obtained SPM data are also compatible within error bars with this linear fit. A satisfying agreement is also found for WEM except for two data points. Furthermore, Fig. 2 also presents DFT-MD calculations. For the water-methane and the water-methane-ammonia mixtures, the LMA has been used as discussed above. We find very good agreement between the experimental data and the simulations, suggesting a validation of the LMA approach along the Hugoniot curve.

Using the Rankine-Hugoniot equations, we extracted the *ρ*-*p*-(*E* − *E*_0_) thermodynamic conditions from the *U*_*s*_-*U*_*p*_ relation. *ρ*-*p*-(*E* − *E*_0_) results are shown in Table 1. Pressure as a function of compression is shown on the bottom of Fig. 2 (see also the Supplementary information for a comparison with the calculations).

**Table 1:** Experimental data on pure water, WEM and the SPM.

Run-sample	${{\boldsymbol{U}}}_{{\boldsymbol{s}}}^{{\bf{Qz}}}$ (km/s)	${{\boldsymbol{U}}}_{{\boldsymbol{s}}}^{{\bf{s}}{\bf{a}}{\bf{m}}{\bf{p}}{\bf{l}}{\bf{e}}}$ (km/s)	*ρ* (g/cm^3^)	*p* (Mbar)	*E* − *E*_0_ (kJ/g)
GK-H_2_O	12.96 ± 0.27	13.91 ± 0.30	2.74 ± 0.18	1.23 ± 0.05	39.2 ± 2.7
L1-H_2_O	18.19 ± 0.31	20.24 ± 0.35	3.00 ± 0.17	2.73 ± 0.07	91.3 ± 4.1
GK-WEM	14.27 ± 0.26	15.80 ± 0.31	2.48 ± 0.15	1.42 ± 0.04	51.9 ± 2.7
GK-WEM	10.73 ± 0.27	11.75 ± 0.30	2.24 ± 0.20	0.74 ± 0.03	25.5 ± 2.0
GK-WEM	18.27 ± 0.26	20.82 ± 0.30	2.65 ± 0.13	2.55 ± 0.06	96.8 ± 3.8
GK-WEM	13.03 ± 0.26	14.60 ± 0.30	2.32 ± 0.14	1.16 ± 0.04	41.1 ± 2.6
GK-WEM	11.94 ± 0.26	12.99 ± 0.30	2.38 ± 0.22	0.94 ± 0.04	33.5 ± 2.7
L1-WEM	18.74 ± 0.27	20.93 ± 0.31	2.86 ± 0.19	2.67 ± 0.07	104.8 ± 5.1
L2 -WEM	18.65 ± 0.41	22.47 ± 0.48	2.37 ± 0.16	2.79 ± 0.11	99.6 ± 6.8
L2-WEM	15.72 ± 0.43	18.37 ± 0.47	2.31 ± 0.19	1.84 ± 0.08	64.8 ± 5.3
GK-SPM	15.49 ± 0.26	17.26 ± 0.30	2.56 ± 0.19	1.73 ± 0.06	63.6 ± 4.0
GK-SPM	15.07 ± 0.26	16.16 ± 0.30	2.78 ± 0.19	1.58 ± 0.05	60.4 ± 3.4
GK-SPM	18.46 ± 0.26	20.71 ± 0.31	2.79 ± 0.23	2.60 ± 0.08	99.4 ± 5.9
GK-SPM	9.72 ± 0.27	10.71 ± 0.30	2.14 ± 0.19	0.60 ± 0.03	19.5 ± 2.1
GK-SPM	10.20 ± 0.27	11.27 ± 0.30	2.18 ± 0.19	0.67 ± 0.03	22.3 ± 2.1
L2-SPM	14.94 ± 0.42	16.29 ± 0.47	2.65 ± 0.31	1.57 ± 0.09	58.5 ± 5.9
L2-SPM	16.58 ± 0.46	19.20 ± 0.52	2.44 ± 0.30	2.08 ± 0.10	74.4 ± 7.8

The run prefixes GK, L1, and L2 identify the campaign at GEKKO XII and the first and second campaign at LULI2000, respectively. The *ρ*-*p*-(*E* − *E*_0_) data are relative to the sample under study.

**Figure 3:**
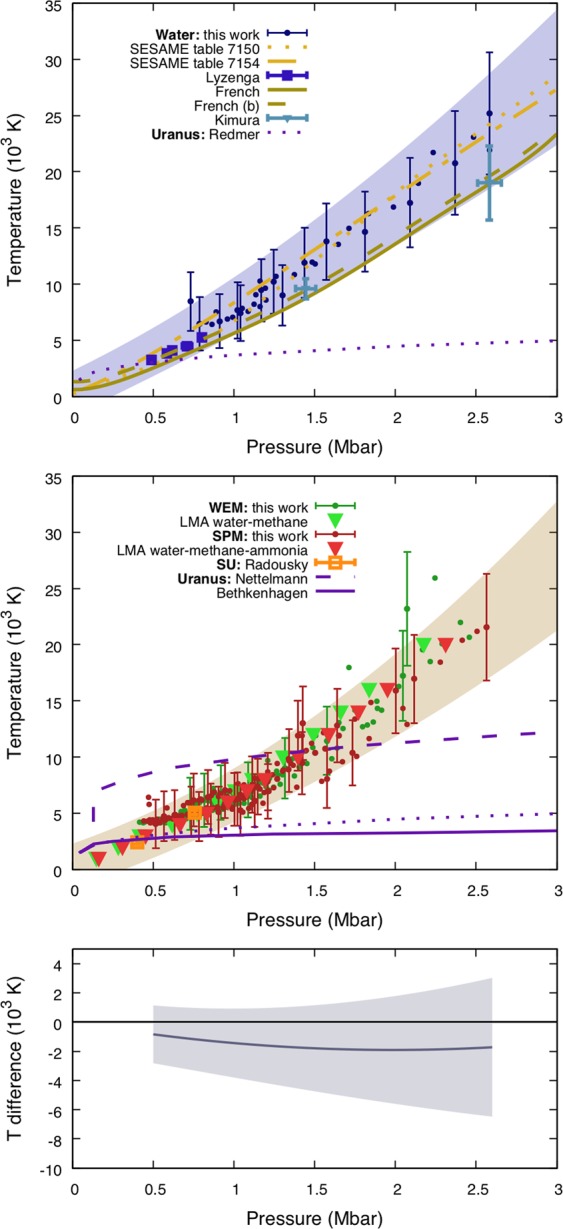
From top to bottom: temperature - pressure relation for pure water (blue data) along the principal Hugoniot curve; the same relation for the WEM (green data) and the SPM (red data); temperature difference between the fit on mixtures and the fit on pure water. Each filled circle is a time-resolved measure during the propagation of a decaying shock. Each area corresponds to the error bars of the associated fit. Previous studies of pure water^24,27,46,54^ and synthetic Uranus^21^ are shown for comparison. Different Uranus interior models^20,52,53^ are shown in purple.

The temperature-pressure (*T*-*p*) relations of water and the considered mixtures are shown in Fig. 3, together with different temperature-pressure profiles predicted for Uranus^20,52,53^. Our data have been fitted with the quadratic function $T(p)={T}_{{\rm{amb}}}+{c}_{1}p+{c}_{2}{p}^{2}$ (with ${T}_{{\rm{amb}}}=300\,{\rm{K}}$). An extrapolation of our fit to lower pressures is compatible with previous gas-gun data^24^. While our data agree within the errors with recent laser shock results^27^, our temperatures are higher than those given by DFT-MD simulations^54^, although the discrepancy is reduced when quantum corrections from molecular vibrations are taken into account^46^ (the predicted temperatures increase of $\simeq$700 K). Our SPM results agree with a previous low-pressure experimental study of the very similar synthetic Uranus^21^. Although the relatively large error bars on temperature make the detection of possible discrepancies rather difficult, water, the WEM, and the SPM temperatures along the principal Hugoniot curves are comparable. We also notice that these findings are in agreement with our DFT-MD calculations using the LMA.

The similar *U*_*s*_-*U*_*p*_ and *T*-*p* relations between water and mixtures can be interpreted in light of previous DFT-MD simulations^5^, which identify the regime explored in this study as an electronically conducting phase. At these temperatures, carbon-carbon and carbon-nitrogen bond lifetimes are predicted to be very short^19^. Therefore phenomena like polymerisation and clustering are not expected to occur and the presence of carbon and nitrogen atoms should not influence the *U*_*s*_-*U*_*p*_ and *T*-*p* relation of the SPM with respect to that of pure water. However, potentially existing chemical processes in the compressed bulk material could not be observed in this study, which relies on shock-front measurements. All over the studied range, our *U*_*s*_-*U*_*p*_ and *T*-*p* data show very good agreement with LMA calculations, which validates this approximation at planetary conditions^20^.

The *T*-*p* data of this work are hereafter compared with the available models of planetary internal profiles. Most of them^20,52^ predict an adiabatic profile inside the icy giants, implying that temperatures stay relatively low (3000–4000 K) even at the highest pressures we explored. Nevertheless, adiabatic models are not consistent with Uranus‘ thermal evolution and more advanced internal structure models including a thermal boundary layer are suggested^53^. The internal profiles hence obtained are much hotter, spanning from 7000–14000 K. Our data therefore bring useful input for these models up to 1.5 Mbar. Moreover, the recent discovery of a large amount of exoplanets exhibits a wide range of structures including hot Neptunes, whose internal profiles can match the thermodynamical conditions we explored. Additionally, our data represent useful constraints for the validation of DFT-MD simulations at extreme conditions.

### Shock-front reflectivity

**Figure 4:**
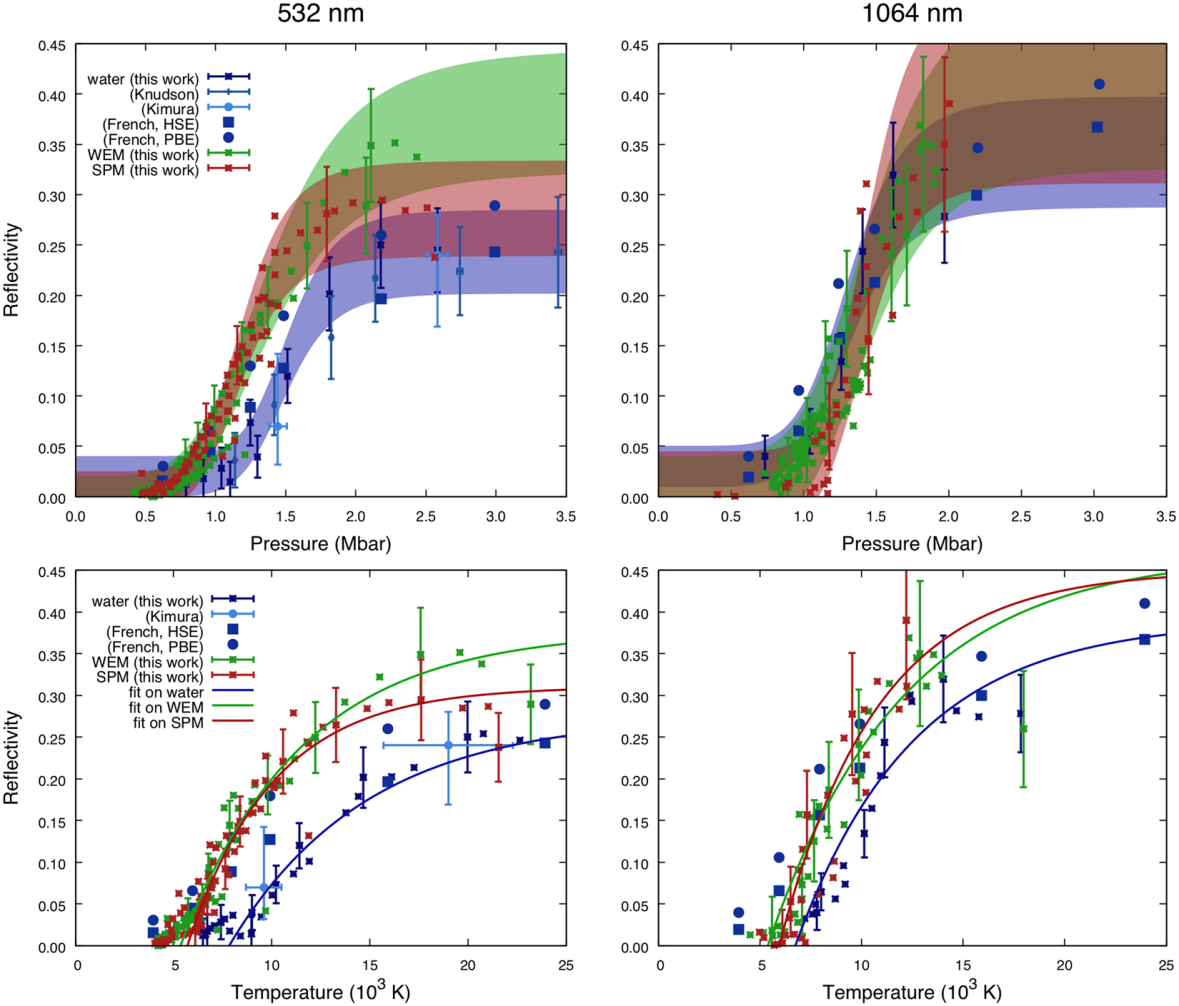
Reflectivity of water and the SPM at 532 nm (left) and 1064 nm (right) as a function of pressure (top) and temperature (bottom) along the principal Hugoniot curves. Top. Reflectivity vs pressure data. Blue, green, and red areas correspond to the fit of our data for pure water, WEM, and SPM within the error bars, respectively. Crosses are time-resolved measures during decaying shock propagation. Filled squares or circles correspond to previous DFT reflectivity calculations^55^ using the HSE and PBE exchange-correlation functionals, respectively. Previous data on water are shown for comparison^26,27^. Error bars are larger at 1064 nm because of the smaller number of available shots. Bottom. Reflectivity vs temperature data. Solid lines serve as guides to the eye.

The optical reflectivities *R* of the water, the WEM, and the SPM shock front at 532 nm and 1064 nm as a function of pressure (top) and temperature (bottom) are shown in Fig. 4. These are the first reflectivity measurements of this type of mixtures; moreover, no calculations of shock-compressed icy mixtures reflectivity exist in the literature to date. For all liquids, the gradual increase of reflectivity, from a low initial value of a few percent up to a saturation value of some ten percent, along the principal Hugoniot indicates a smooth transition from an insulating to an electronically conducting (“metallic”) state with the increase of density, pressure, and temperature.

We performed a fit on each *R*-*U*_*s*_ relation using a Hill function $R({U}_{s})={R}_{0}+({R}_{sat}-{R}_{0})\cdot {U}_{s}^{k}$/$({U}_{s}^{k}+{U}_{0}^{k})$, which is suitable to model this gradual transition. The error bars of the fit are discussed in the Supplementary information and mainly depend on the calibration. The function has then been rescaled to be pressure-dependent using an experimentally determined equation of state for water^26^ and a fit on our data for the WEM and the SPM to link pressure and shock velocity. On the left we present a direct comparison between water, SPM and WEM at 532 nm while on the right the probe is at 1064 nm. Strong differences are observed in particular at 532 nm. The main one concerns the onset of reflectivity occurring in water at 1.1–1.2 Mbar while in SPM and WEM is observed at lower pressures, around 0.8 Mbar. The saturation values are also different (24% for water against 29% in SPM and even higher in WEM). Concerning water, our 532 nm reflectivity data are in quantitative agreement with previous experiments^26,27^. When compared with existing calculations^55^, at 532 nm, our reflectivity data suggest that the HSE exchange-correlation functional is more accurate than PBE, confirming what previous observations^26^. At 1064 nm, the differences are less important, which indicates a complex behaviour of optical conductivity that needs to be investigated with theoretical calculations in the future. We note that saturation values of reflectivity at 1064 nm for both liquids are greater than those at 532 nm. Water data at 1064 nm are in qualitative agreement with the calculations. Reflectivity data are also presented as a function of temperature (bottom of Fig. 4) showing a similar trend.

The fact that the onset pressure of the SPM and the WEM reflectivity at 532 nm occurs at pressures around 26% lower than in water cannot be fully explained by the 12% difference between their initial densities. The higher reflectivity of the mixtures may be due to a higher free electron density at the same thermodynamic conditions, as the ionisation energy of carbon-bearing molecules (*e*.*g*. 10.5 eV for ethanol) is lower than that of water (12.6 eV)^56^. This hypothesis seems to be corroborated by the reflectivity saturation values at 532 nm. Indeed they are 35% for the WEM, 29% for the SPM, and 24% for water, consistently ordered by carbon concentration.

Reflectivity data are essential due to their connection to electronic conductivity. In the literature, the extraction of the electronic conductivity from reflectivity data is often obtained using a classic or modified Drude model^25,28^. The assumptions of the Drude model in this regime are questionable, as water and mixtures are known not to follow a Drude behaviour for what concerns the frequency dependence of electronic conductivity. Nonetheless, it is insightful to use it to compare the conductivity water and the SPM. This way we estimate conductivity saturation values of 2200 and 3400 S/cm for water and SPM, respectively. The details of this estimation are presented in the Supplementary information.

## Conclusions

In this work, pure water, a water-ethanol mixture (WEM), and a water-ethanol-ammonia “synthetic planetary mixture” (SPM) have been compressed by laser-driven decaying shocks up to a pressure of 2.73 ± 0.07, 2.79 ± 0.11, and 2.60 ± 0.08 Mbar, respectively. The thermodynamic states and shock-front reflectivities at 532 nm and 1064 nm have been measured along their principal Hugoniot curves. The datasets presented in this work significantly extend the previously reported results on icy planetary mixtures and provide constraints for theoretical discussions, *e*.*g*. by DFT-MD simulations.

The shock-fluid velocity relations for water, the WEM, and the SPM are comparable within the error bars. Their temperature-pressure relations are also comparable, although possible small discrepancies cannot be ruled out due to the error bars on the fitted relations. The performed LMA calculations of the Hugoniot curves of a water-methane and a water-methane-ammonia mixture suggest that the experimental data validate such an approach.

Pure water and mixtures exhibit different shock-front reflectivity behaviours. The pressure onsets of reflectivity for the WEM and the SPM are lower than that of water, while the reflectivity saturation values are higher for the SPM and WEM than for water. This may be a signature of the fact that carbon-rich mixtures dissociate at lower pressures and temperatures than pure water. Our data constitute thus a touchstone for the DFT-MD calculations of the structural and transport properties for complex mixtures with varying compositions of heavy elements. Finally, our reflectivity data show that the electronic conductivity of the SPM is larger than that of water. Such a result can have direct repercussions on planetary models including a thermal boundary layer^53^, where the electronic contribution is predominant with respect to the ionic one, due to the high temperatures involved.

Future experimental work should study high-pressure off-Hugoniot states to enlarge the explored scenario and probe thermodynamic conditions closer to those expected in planetary interiors. Pure liquid methane and pure liquid ammonia should be studied to provide a complete framework of the properties of the icy end-members and their mixtures. Finally, X-ray diffraction studies should be conducted on synthetic planetary ices to probe their bulk structure, thus overcoming the limitations of shock-front measurements.

## Supplementary information

Supplementary Information

## Data Availability

The data that support the plots within this paper and other findings of this study are available from the corresponding authors upon reasonable request.
