# Supplementary material for: Laser-driven shock compression of “synthetic planetary mixtures” of water, ethanol, and ammonia
Source: Sci Rep. 2019 Jul 12;9:10155. doi: 10.1038/s41598-019-46561-6 (PMC6626017; doi:10.1038/s41598-019-46561-6)
Supplement: Supplementary file 1 — Supplementary Information [file 41598_2019_46561_MOESM1_ESM.pdf]

Supplementary information for "Laser-driven shock compression of "synthetic planetary mixtures" of water, ethanol, and ammonia"

M. Guarguaglini[1, 2, *], J.-A. Hernandez[1, 2], T. Okuchi[3], P. Barroso[4], A. Benuzzi-Mounaix[1, 2], M. Bethkenhagen[5], R. Bolis[1, 2], E. Brambrink[1, 2], M. French[5], Y. Fujimoto[6], R. Kodama[6, 7, 8], M. Koenig[1, 2, 7], F. Lefevre[1], K. Miyanishi[8], N. Ozaki[6, 8], R. Redmer[5], T. Sano[8], Y. Umeda[6], T. Vinci[1, 2], and A. Ravasio[1, 2, +]

[1]LULI, CNRS, CEA, École Polytechnique, Institut Polytechnique de Paris, route de Saclay, 91128 Palaiseau cedex, France
[2]Sorbonne Université, Faculté des Sciences et Ingénierie, Laboratoire d'utilisation des lasers intenses (LULI), Campus Pierre et Marie Curie, place Jussieu, 75252 Paris cedex 05, France
[3]Institute for Planetary Materials, Okayama University, Misasa, Tottori 682-0193, Japan
[4]GEPI, Observatoire de Paris, PSL Université, CNRS, 77 avenue Denfert Rochereau, 75014 Paris, France
[5]Universität Rostock, Institut für Physik, 18051 Rostock, Germany
[6]Graduate School of Engineering, Osaka University, Suita, Osaka 565-0871, Japan
[7]Open and Transdisciplinary Research Initiatives, Osaka University, Suita, Osaka 565-0871, Japan
[8]Institute of Laser Engineering, Osaka University, Suita, Osaka 565-0871, Japan
[*]marco.guarguaglini@polytechnique.edu
[+]alessandra.ravasio@polytechnique.edu

S1. Target cells

Target cells are made of aluminium and measure $15 \times 15 \times 7.5$ mm. The cylindrical liquid volume in the laser-diagnostics axis has a 2 mm diameter and is 4 mm thick. Two lateral holes, plugged with capillaries, enable the filling of the cell. Target conception has been realised at the Observatoire de Paris. The ablator/pusher combination (CH / Al / (Au) / SiO_2) has been glued on the drive laser side window of the cell, while a SiO_2 window has been glued on the diagnostics side. Thickness of glue was around 1μm. The plastic we used was polystyrene. The two copper or aluminium capillaries of the target cell have been glued onto the corresponding holes of the cell and connected to plastic tubes (see Figure S1). The mixture has then been injected in the cell through air suction using a pump (GEKKO experiment) or a syringe (LULI experiment). Finally, the capillaries have been cut making the cell globally watertight.

S2. VISARs

VISARs (Velocity Interferometry System for Any Reflector) can measure the velocity of a reflective shock front U_s from a Doppler-shift induced fringe displacement, according to the formula:

$$U_s = \frac{\lambda_0}{2\tau(1+\delta)n_0} \cdot \frac{\Delta\phi}{2\pi}, \tag{S1}$$

where λ_0 is the probe laser wavelength, τ is the delay induced by a transparent etalon placed on one of the two arms of the interferometer, δ is the etalon dispersion, n_0 is the pristine material refractive index, and

$\Delta\phi$ the phase shift on the fringe system.
The velocity per fringe parameters VPF $= \lambda_0/2\tau(1+\delta)$ of the VISARs were 4.476 and 7.432 km/s (GEKKO XII) or 15.94 and 6.08 km/s (ω and 2ω VISARs at LULI2000, respectively).
The use of at least two VISARs with different VPF is mandatory since, if a single VISAR is used, $\Delta\phi$ can only be determined modulo 2π. A quantity $2m\pi$ must thus be added to the measured phase shift. This quantity can be found by imposing the superposition of the two VISAR velocities.
The VISARs signals were injected to streak cameras whose time range were 20 or 50 ns. The slit opening was 50 or 100 μm.

S3. SOP calibration

At GEKKO XII, the SOP has been calibrated with the use of shocked quartz as standard, using the measured shock velocity and self-emission and a previously established T-U_s relation[1]. At LULI2000, the calibration has been made with the use of a standard lamp (OSRAM Wi 17/G) with known emission temperature. The streak camera has been fired 50 times for each time window from 1 ms to 10 ns and a statistical mean value of the number of counts N_c has been extracted. A linear fit on the time window-dependent number of counts $N_c(\Delta t) = a\,\Delta t + b$ (where b is the intrinsic background) allowed to determine the calibration factor A:

$$A = \left(e^{T_0/T_{lamp}} - 1\right) a\,\Delta t, \tag{S2}$$

where $T_{lamp} = 2610$ K is the emission temperature of the lamp.

S4. Error estimation

The error on the measured fringe shift on the VISAR output has been estimated as $1/10$ of a fringe. The error on U_s thus depended on the number of the 2π shifts added to the measured shift to superimpose the two VISAR signals. A typical error on U_s was 3%. A Monte Carlo routine has been implemented to propagate the errors through the impedance mismatching analysis (as shown in Figure S2). Error sources were the error on U_s^{Qz} and U_s^{mix} (from the VISAR measurement and from the linear fit) and the uncertainty on the quartz Hugoniot. Each analysis run used random input from a Gaussian distribution.
The error on reflectivity has different sources. The main one depends on the calibration method. The fit operated on quartz measurements has a typical relative error of about 15%. Another source derives from the background estimation of the reference and the shot image. An uncertainty of the background value propagates when background is subtracted from the value extracted from the VISAR image. This introduces a typical relative error of about 2%, weakly dependent on the reflectivity value.
Temperature is obtained from both SOP and VISAR measurements (of emission intensity and emissivity, respectively). The main error sources are the SOP calibration factor, the error on the number of SOP counts, and the error on VISAR-measured reflectivity. The use of the emission to determine the temperature induces the existence of a detection limit. The detection limit temperature corresponds to a number of SOP counts that is comparable to the uncertainty on this value: $N_c \simeq \Delta N_c$. It can be expressed as:

$$T_{d.l.} \simeq \frac{T_0}{\ln\left(A/\Delta N_c\right)}. \tag{S3}$$

Typical detection limit temperatures are about 4000 K. Typical errors on the temperature are $\sim 20\%$, far enough from the detection limit.

S5. Study of pure water

Figure S3 shows an example of diagnostics output for a shot on pure water at the LULI2000 facility. The shock - particle velocity relation along the principal Hugoniot curve of pure water is shown in Figure S4. The density at ambient conditions is $\rho_0^{\text{water}} = 0.998$ g/cm^3 and the refractive index at 532 nm and 1064 nm are 1.3337 and 1.3260, respectively[2]. For $U_p > 7$ km/s, data in the literature do not universally agree, as the SESAME table 7154 and the work by Henry *et al.*[3] find a lower slope than more recent work[4,5]. Though our low-pressure datum cannot discriminate between the two slopes, our high-pressure datum clearly agrees with a linear fit on data by Knudson *et al.*[4] and is not compatible with the SESAME table.

S6. Comparison between calculations and experimental data

Figure S5 shows the adjusted pressure p/ρ_0 over the compression factor ρ/ρ_0 along the measured and DFT-MD Hugoniot curves of water and the different mixtures. The scaling of both axes with the initial densities ρ_0 is performed to account for the different compositions of the various mixtures. The initial densities of the Hugoniot curves derived from DFT-MD are 0.998 g/cm^3 for pure water, 0.7301 g/cm^3 for the water-methane-ammonia mixture, and 0.7019 g/cm^3 for the water-methane mixture at a initial temperature of 298 K. Note, that the initial DFT-MD densities and energies of the water-methane and water-methane-ammonia mixtures are chosen such that they correspond to the initial densities of WEM and SPM, respectively.

Overall, we find remarkable agreement between the experimental data from present as well as previous work[4–7] and the present DFT-MD Hugoniot for water, whereas there are some discrepancies between experiment and simulation for the mixtures. Our simulations for the mixtures predict compression ratios similar to that of water up to an adjusted pressure of 0.8 Mbar cm^3/g. For adjusted pressures beyond this point, the DFT-MD Hugoniot curves predict significantly higher compression factors compared to pure water. In this range, the compression ratio calculated for the water-methane mixture is found slightly higher compared to the ternary water-methane-ammonia mixture. This systematic compression behaviour is not as evident in the experimental data, but we also find the measured compression ratios for the mixtures to be higher than for water except for a few points. This difference between experiment and calculations is most likely due to the slightly different hydrogen ratios considered in the mixtures in the calculations. The water-methane-ammonia and the water-methane mixtures have atomic ratios of H:C:N:O = 33:4:1:7 and H:C:N:O = 30:4:0:7, respectively, while the ethanol mixtures in the experiment have ratios of H:C:N:O = 25:4:1:7 and H:C:N:O = 22:4:0:7. Although this difference in hydrogen content has little effect on the pressure-temperature relation as shown in Fig. 3 in the main manuscript, the compression ratios are more sensitive to this deviation. This behaviour results from the fact that the hydrogen compression ratios are significantly higher at a given pressure as illustrated in Figure S6. In this plot, we show exemplarily the water, methane, and hydrogen Hugoniot curves corresponding to the linearly-mixed water-methane Hugoniot. The initial densities are 0.9377 g/cm^3 for water, 0.4696 g/cm^3 for methane, and 0.1019 g/cm^3 for hydrogen. Since our LMA water-methane mixture is rich in hydrogen compared to the experimentally investigated water-ethanol mixture, our simulations predict systematically too high compression ratios. If the calculated Hugoniot curves were based on a less hydrogen-rich mixture, the calculated mixture points in Figure S5 would shift to lower compression ratios. However, this does not affect our conclusions on the applicability of the LMA as can be seen by comparing both water curves in Figure S6. An adjusted hydrogen content would move all solid lines slightly to the left in this plot towards the water Hugoniot with the initial experimental density of 0.998 g/cm^3. Both red curves would exactly agree, if the LMA would work perfectly. Therefore, our calculations containing methane instead of ethanol give an upper

bound for estimating discrepancies between real mixtures and the LMA.

S7. Estimation of the electrical conductivity

The electrical conductivity is one of the key parameters for the understanding of planetary magnetic fields. Indeed, a planetary dynamo can be sustained if magnetic induction dominates over magnetic diffusion. This is usually expressed by the requirement that the magnetic Reynolds number $R_m = \mu_0 \sigma u L \gtrsim 100$ (where σ is the electrical conductivity of the active planetary layer component and u and L are the velocity and length scale of the fluid motion inside the layer, respectively).

In gas-gun experiments, the DC electrical conductivity can be directly measured using electrodes. This approach cannot be applied to laser shock experiments. Instead, they would need a measurement of the complex refractive index of the shocked sample $\tilde{n} = n + ik$ since, from the wave solution of the Maxwell's equations, $\sigma(\omega) = 2\varepsilon_0 n(\omega) k(\omega) \omega$. In a restricted range of pressure and temperature the absorption coefficient $\alpha(\omega) = 2\omega k(\omega)/c$ and the reflectivity

$$R(\omega) = \frac{[n(\omega) - n_0(\omega)]^2 + k^2(\omega)}{[n(\omega) + n_0(\omega)]^2 + k^2(\omega)} \tag{S4}$$

(where n_0 is the real part of the un-shocked refractive index and the imaginary part $k_0 = 0$ as is the case for initially transparent materials) can be simultaneously measured[8] at a probe laser wavelength ω. In this case, the evaluation of the conductivity is straightforward. Nevertheless, this approach is very delicate and remains restricted to few experiments and conditions. In laser shock experiments only reflectivity at one or two probe laser wavelengths (often in the green, at 532 nm, and sometimes in the near infrared, at 1064 nm) is usually measured.

In this case, a model has to be considered in order to infer the complex refractive index and thus the electrical conductivity. A common approach considers a local response regime modified to account for bound charge carriers. Within this context, the complex refractive index is expressed as

$$\tilde{n}(\omega) = \left(\tilde{n}_b^2(\omega) + \frac{i\tilde{\sigma}(\omega)}{\varepsilon_0 \omega} \right)^{1/2}, \tag{S5}$$

where $\tilde{n}_b(\omega)$ is the contribution of the bound electrons to the refractive index at the probe laser frequency and $\tilde{\sigma}(\omega)$ is the electrical conductivity at that frequency. The DC conductivity $\sigma(0)$ is often[1,8–10] estimated assuming that the studied material follows a Drude behaviour, *i.e.* that the frequency dependency of the conductivity can be written as

$$\tilde{\sigma}(\omega) = \frac{1}{1 - i\omega\tau} \sigma(0), \tag{S6}$$

where τ is the electron-ion scattering time. However, this hypothesis introduces another unknown, the relaxation time τ, which has to be estimated depending on the thermodynamic conditions. Moreover, although no DFT-MD calculations of the frequency dependence of the conductivity are available in the literature for water or planetary mixtures, studies on other materials[11,12] have shown that a Drude-like behaviour is unlikely to be followed for temperatures lower than several ten thousand of Kelvin. We made the simplistic assumption that the conductivity dependence on frequency can be neglected from the DC value to the near-infrared and visible range: $\sigma(0) \simeq \sigma(\omega_L) \simeq \sigma(2\omega_L)$, where ω_L is the frequency corresponding to 1064 nm (thus $2\omega_L$ corresponds to 532 nm). To model the bound electrons contribution the the refractive index n_b for the explored thermodynamic conditions, we supposed that it remains real

($\tilde{n}_b(\omega) = n_b(\omega)$) and that it can be expressed by extrapolating a Gladstone-Dale model valid for water in the visible range[13]. We also neglected the wavelength dependence of n_b between 532 nm and 1064 nm. Although this model has major limitations, it can be used to compare our data with previous data obtained with the same approach. In Figure S7 the estimated DC conductivity of water and the SPM are shown as a function of temperature. The conductivity of water appears to increase with temperature and reach a plateau around 15000 K. The estimated profile of the SPM is available for temperatures that already correspond to a quasi-saturation behaviour. At 20000 K, conductivity values are $\sim 2.2 \cdot 10^3$ S/cm and $\sim 3.4 \cdot 10^3$ S/cm for water and the SPM, respectively. The estimated conductivity of the SPM is greater than that of water in the entire explored temperature range. A different behaviour was observed in multiple shock experiments on "synthetic Uranus"[14,15]. These experiments probed colder conditions where the main contribution to the conductivity is ionic, whereas in our case the dominant contribution is electronic. This conductivity estimation highlights that the use of the transport properties of water as representative of the planetary ices of Uranus and Neptune for modelling their magnetic dynamos may be too simplistic and incorrect.

References

1. Millot, M. *et al.* Shock compression of stishovite and melting of silica at planetary interior conditions. *Sci.* **347**, 418–420 (2015).
2. Hale, G. M. & Querry, M. R. Optical constants of water in the 200-nm to 200-μm wavelength region. *Appl. Opt.* **12**, 555–563 (1973).
3. Henry, E. *Équation d'état et métallisation de l'eau comprimée par choc laser*. Ph.D. thesis, École Polytechnique (2003).
4. Knudson, M. *et al.* Probing the interiors of the ice giants: Shock compression of water to 700 GPa and 3.8 g/cm^3. *Phys. Rev. Lett.* **108**, 091102 (2012).
5. Kimura, T. *et al.* P-ρ-T measurements of H_2O up to 260 GPa under laser-driven shock loading. *J. Chem. Phys.* **142**, 164504 (2015).
6. Mitchell, A. & Nellis, W. Equation of state and electrical conductivity of water and ammonia shocked to the 100 GPa (1Mbar) pressure range. *J. Chem. Phys.* **76**, 6273–6281 (1982).
7. Lyzenga, G. A., Ahrens, T. J., Nellis, W. J. & Mitchell, A. C. The temperature of shock-compressed water. *J. Chem. Phys.* **76**, 6282–6286 (1982).
8. Millot, M. *et al.* Experimental evidence for superionic water ice using shock compression. *Nat. Phys.* **14**, 297–302 (2018).
9. Celliers, P. M. *et al.* Electronic conduction in shock-compressed water. *Phys. Plasmas* **11**, L41–L44 (2004).
10. McWilliams, R. S. *et al.* Phase transformations and metallization of magnesium oxide at high pressure and temperature. *Sci.* **338**, 1330 – 1333 (2012).
11. Laudernet, Y., Clérouin, J. & Mazevet, S. Ab initio simulations of the electrical and optical properties of shock-compressed SiO2. *Phys. Rev. B* **70**, 165108 (2004).
12. Clerouin, J., Renaudin, P., Laudernet, Y., Noiret, P. & Desjarlais, M. P. Electrical conductivity and equation-of-state study of warm dense copper: Measurements and quantum molecular dynamics calculations. *Phys. Rev. B* **71**, 064203 (2005).

13. Batani, D. *et al.* Refraction index of shock compressed water in the megabar pressure range. *Europhys. Lett.* **112**, 36001 (2015).

14. Nellis, W., Holmes, N., Mitchell, A., Hamilton, D. & Nicol, M. Equation of state and electrical conductivity of synthetic Uranus, a mixture of water, ammonia, and isopropanol, at shock pressures up to 200 GPa. *J. Chem. Phys.* **107**, 9096–9100 (1997).

15. Chau, R., Hamel, S. & Nellis, W. Chemical processes in the deep interior of Uranus. *Nat. Commun.* **2**, 203 (2011).

16. Radousky, H., Mitchell, A. & Nellis, W. Shock temperature measurements of planetary ices: NH_3, CH_4, and synthetic Uranus. *J. Chem. Phys.* **93**, 8235–8239 (1990).

17. French, M., Mattsson, T. & Redmer, R. Diffusion and electrical conductivity in water at ultrahigh pressures. *Phys. Rev. B* **82**, 174108 (2010).

18. French, M. & Redmer, R. Electronic transport in partially ionized water plasmas. *Phys. Plasmas* **24**, 092306 (2017).

Figures

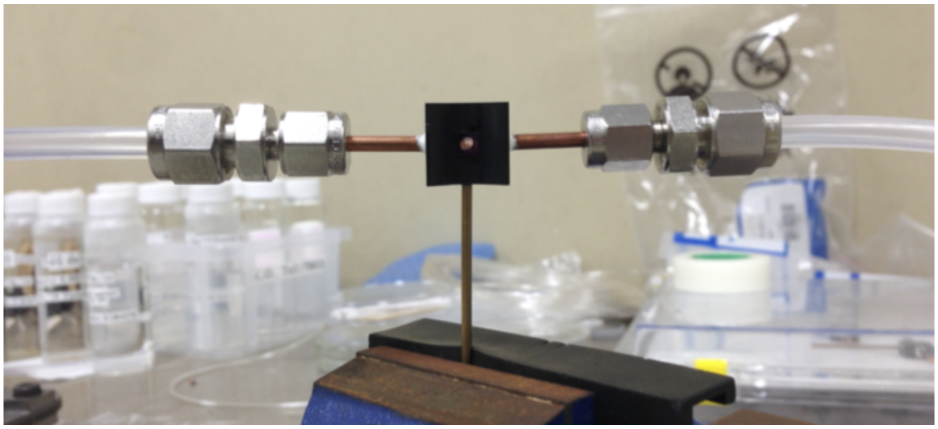

Figure S1. Target cell with copper capillaries connected to the plastic tubes for filling.

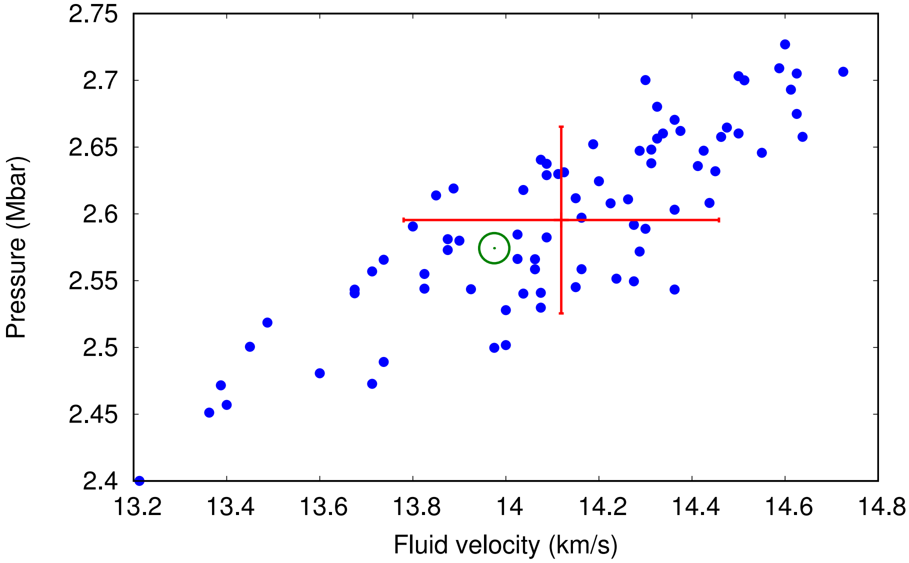

Figure S2. Monte Carlo error analysis in the (U_p, p) plane for a SPM equation of state point. Blue circles are the results of each single run of the analysis, for varying U_s^{Qz} and U_s^{mix} around a central value. The red cross with error bars represents the mean value with the standard deviation. The green circle is the result obtained from the central value of the measure.

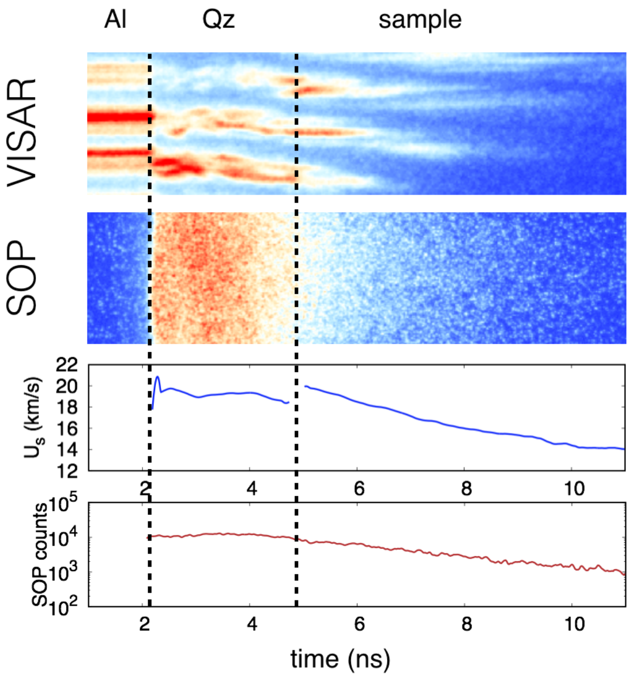

Figure S3. Diagnostics output of a typical shot on pure water at the LULI2000 facility. From top to bottom: 1064 nm VISAR image; SOP image; time-resolved shock velocity; time-resolved SOP counts. The three time intervals indicate when the probe laser is reflected by aluminium (Al), when a reflecting shock front is propagating through the quartz layer (Qz), and when the shock front is propagating through the mixture sample.

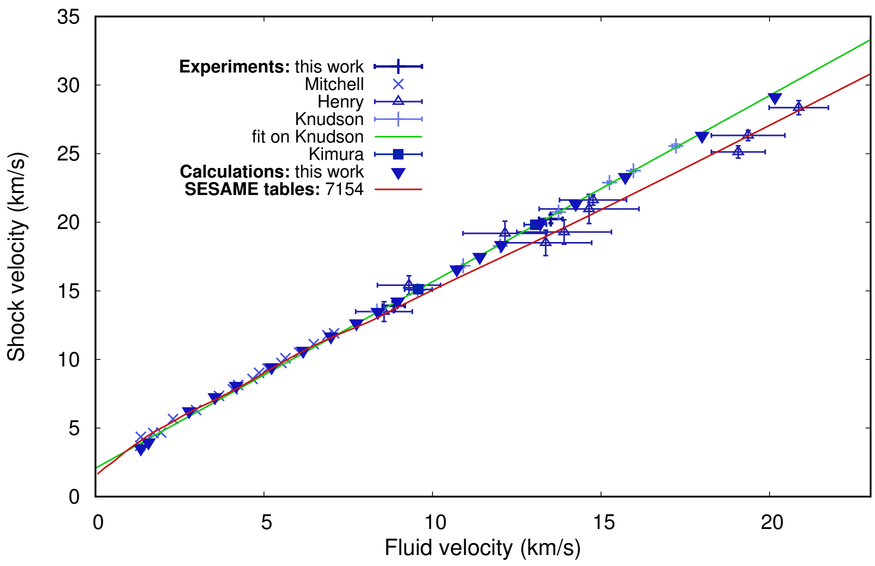

Figure S4. Shock vs particle velocity relation for water. Data from our two shots and the calculations performed in this work are shown together with results from previous studies[3–6]. Additionally, the relation extracted from the SESAME table 7154 for water is shown.

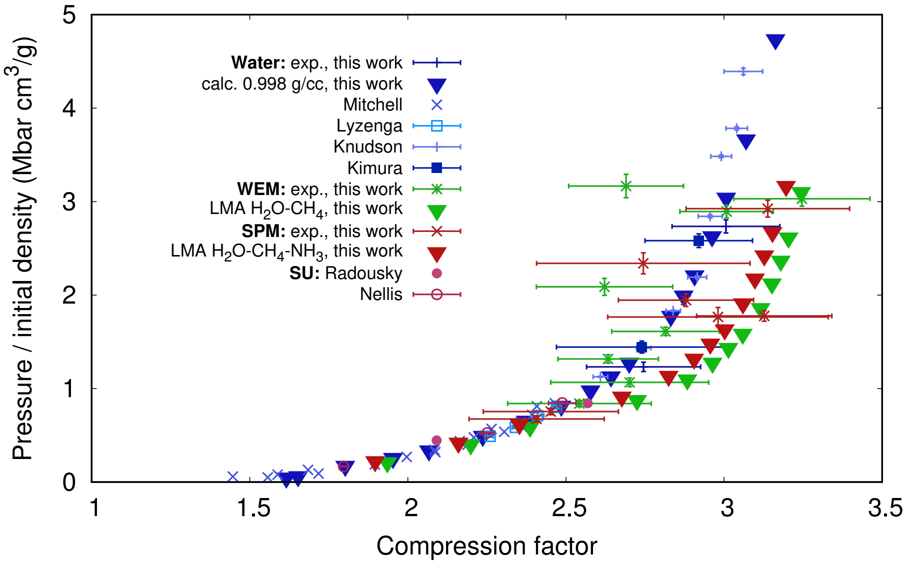

Figure S5. Adjusted pressure p/ρ_0 over compression factor ρ/ρ_0 from DFT-MD data (solid triangles) in comparison to experimental data from this work and previous work[4–7,14,16]. Water data are presented in blue/cyan, while binary and ternary mixtures are green and red/pink, respectively.

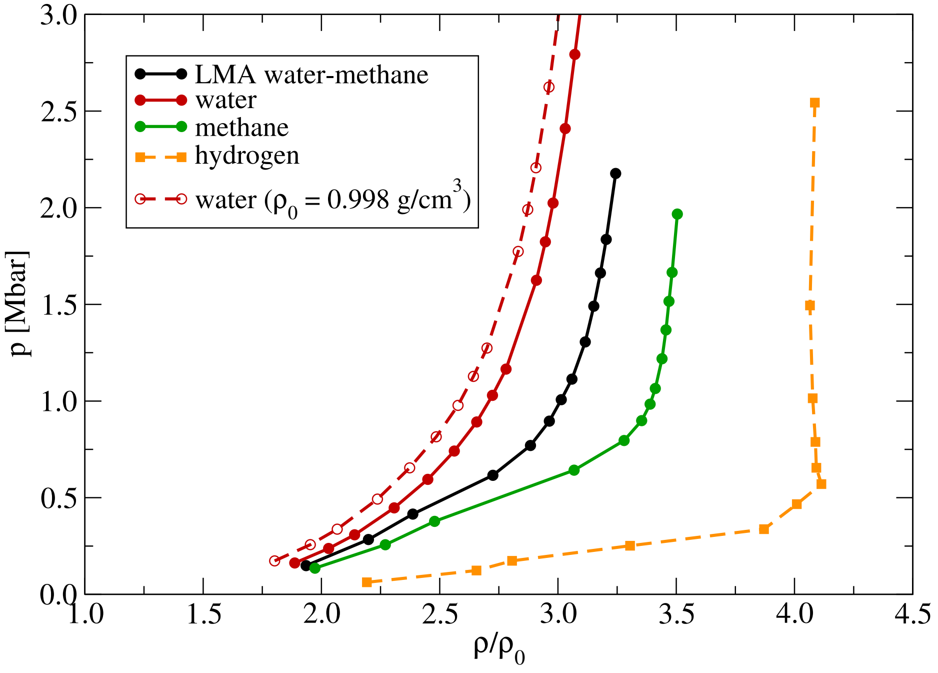

Figure S6. Hugoniot curves corresponding to the initial conditions of the experimentally investigated water-ethanol mixture. The LMA Hugoniot for the 7:4 water-methane mixture (solid black line) was constructed using the water and methane EOS described in the text, whose Hugoniot curves are given as solid red and green lines, respectively. The dashed red line corresponds to the experimental pure water data presented in this work. The Hugoniot of hydrogen (dashed yellow line) is given to illustrate the effect of the excess of hydrogen contained in the 7:4 water-methane mixture compared to the experimentally investigated water-ethanol mixture.

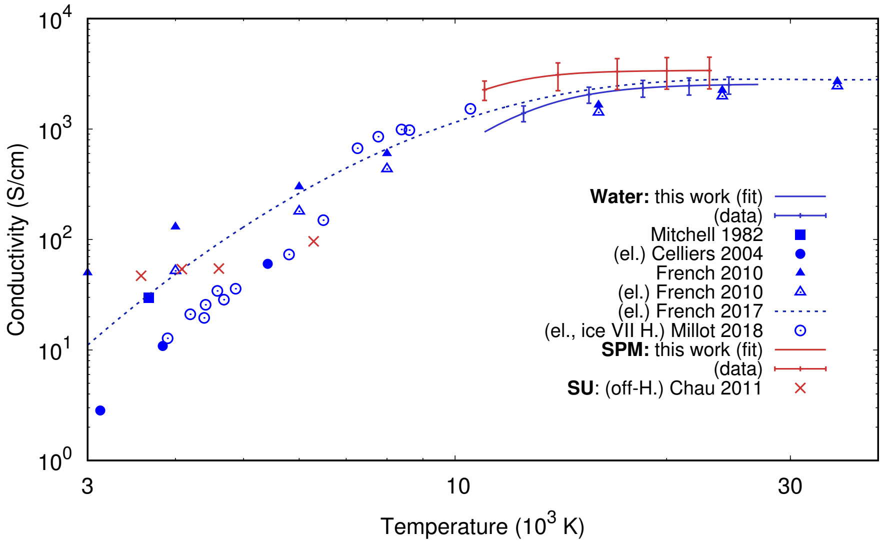

Figure S7. Estimated DC electrical conductivity of water and the SPM as a function of temperature. Data in the literature for water[6,8,9,17] and of "synthetic Uranus"[15], similar to the SPM, are shown for comparison. The reduction along the principal Hugoniot of a fit on DFT-MD calculations of the water conductivity as a function of density and temperature[18] is also shown (blue dashed line). All data are relative to the principal Hugoniot curve of the material, except when explicitly marked ("ice VII H." means that the data are along the water Hugoniot starting from ice VII at $\rho_0 = 1.6$ g/cm^3; "off H." means that data are off the principal Hugoniot since the conditions have been obtained via multiple shocks). The data show total (electronic + ionic) conductivity, unless explicitly marked ("el." means that only the electronic contribution is shown).
